# Total anomalous pulmonary vein drainage: Report of an autopsy case associated with atresia of the common pulmonary vein and left superior pulmonary vein

**DOI:** 10.1111/j.1440-1827.2010.02617.x

**Published:** 2011-02

**Authors:** Sohsuke Yamada, Masanori Hisaoka, Ke-Yong Wang, Yan Ding, Xin Guo, Shohei Shimajiri, Hayato Matsumoto, Yoshitsugu Shirakawa, Yasuyuki Sasaguri

**Affiliations:** 1 Department of Pathology and Cell Biology, School of Medicine, University of Occupational and Environmental HealthKitakyushu, Japan; 2 Department of Pathology and Oncology, School of Medicine, University of Occupational and Environmental HealthKitakyushu, Japan; 3 Department of Pediatrics, Fukuoka Shin Mizumaki HospitalKitakyushu, Japan

**Keywords:** atresia of the common pulmonary vein (ACPV), atresia of the left superior pulmonary vein, neonate, pulmonary lymphangiectasis (PL), total anomalous pulmonary vein drainage (TAPVD)

## Abstract

We describe the clinicopathological features of a case of total anomalous pulmonary vein drainage (TAPVD) associated with atresia of the common pulmonary vein (ACPV). A male Japanese infant born at 37 weeks of gestation demonstrated apnea and severe respiratory acidosis immediately after delivery. The patient died of hypoxemic respiratory failure 6 days after birth despite the initiation of artificial ventilation and administration of a surfactant. Autopsy showed the bilateral inferior pulmonary veins joined with a blind confluence, representing ACPV, accompanied by atresia of the left superior pulmonary vein. Moreover, the anomalous and small right superior pulmonary vein drained into the superior vena cava, consistent with partial and supracardiac type TAPVD. A histological examination of the lungs exhibited diffuse dilation of the lymphatic channels in the peribronchial, interlobular, hilar and focally, subpleural areas. The channels were lined with flattened endothelium which was immunohistochemically positive for D2-40. These findings conformed to a secondary form of pulmonary lymphangiectasis due to the congenital cardiovascular anomalies, including TAPVD and ACPV. To the authors' knowledge, this is the first case of TAPVD associated with ACPV, atresia of left superior pulmonary vein and pulmonary lymphangiectasis.

Atresia of the common pulmonary vein (ACPV) is an extremely rare congenital cardiac defect and a poorly documented disease in neonates, it is characterized by the abnormal left and right pulmonary veins (PV) failing to communicate with either the left and right atriums or any of the major systemic veins.^1^,^2^ The common pulmonary vein (CPV) is an embryonic structure connecting the venous elements of early pulmonary vasculature to the common atrium and is subsequently incorporated as part of the left atrium occurring between the 25^th^ and 30^th^ day of fetal life.^3^ Thus, inadequate or absent absorption induces certain congenital anomalies.

Early atresia of the CPV leads to total anomalous pulmonary venous drainage (TAPVD), a fairly common entity that accounts for 1.3% of congenital cardiovascular malformations and is detected in 2 to 5% of autopsy cases of congenital heart disease.^4^ Darling classified TAPVD into four types by which the systemic venous system drains into the PV or CPV: type I is characterized by supracardiac connections (e.g. the innominate (left brachiocephalic) vein or superior vena cava); type II, paracardiac connections (e.g. the coronary sinus or right atrium); type III, infracardiac connections (e.g. the inferior vena cava, portal vein, hepatic vein, or ductus venosus); type IV, multiple and mixed, with two or more types of I to III.^5^ Drainage to the innominate vein occurs most commonly and is found in over 35% of TAPVD cases, followed by those to the superior vena cava (about 13%), the coronary sinus (16%), the right atrium (15%), and the portal vein (6%).^5^,^6^ In contrast, late atresia or incomplete absorption of the CPV after involution of the embryonic collaterals to the systemic venous circulation leads to ACPV.^1^,^2^,^7^,^8^ At least 32 cases of ACPV have been reported in the literature^8^,^9^ since the term was first used and described by Lucas *et al*. in 1962.^10^ ACPV could be considered an extreme form of TAPVD in which virtually complete PV obstruction occurs in the uterus.^2^ However, this anomaly should be clearly distinguished from the stenosis of aberrant PV at their junction with the systemic circulation common in TAPVD cases after birth or surgery.^6^ Prenatally, the small pulmonary venous blood flow can be diverted to the systemic veins, for example the azygos and hemiazygos veins, by way of bronchopulmonary anastomoses in the lungs,^2^,^10^–^12^ and persistently dilated fetal pulmonary lymphatic channels help to remove the excess interstitial edema fluid from the lungs.^2^,^7^,^13^,^14^ Therefore, the fetus possibly tolerates severe obstruction to pulmonary venous drainage. In contrast, postnatally, with the tenfold increase in pulmonary blood flow, these channels are very easily overwhelmed.^15^ Neonates with ACPV generally display symptoms of cardiorespiratory failure, such as cyanosis and/or acidosis within minutes of birth.^1^,^2^,^6^–^11^ Pulmonary interstitial edema or occasional secondary pulmonary lymphangiectasis (PL) also impairs the gaseous exchange.^2^,^9^,^10^,^16^ Cardiovascular and respiratory collapse and subsequent death rapidly occur in many ACPV cases, and early recognition and surgical intervention are essential to patient care.^1^,^2^,^6^–^11^ According to previous reports, however, only five cases have undergone successful surgical repair.^2^,^7^

In this paper, we describe an autopsy case of TAPVD type I, in which anomalous small right PV drained to the superior vena cava (SVC), associated with ACPV of the left and right inferior PV (IPV) with blind confluence and atresia of the left superior PV (SPV). In addition, secondary PL was also identified in the histological examination.

## CLINICAL SUMMARY

**Figure 1:**
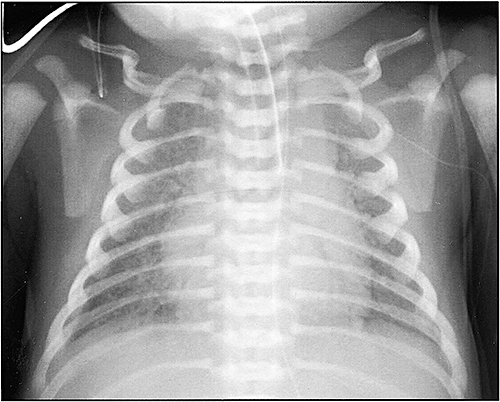
Plain chest X-ray. A chest X-ray film taken just before the patient's death shows marked pulmonary congestion and diffuse bilateral reticulogranular infiltrates. No apparent cardiomegaly is seen.

A Japanese male neonate was born at 37 weeks of gestation without history of pregestational treatment for infertility through Caesarean section due to poor fetal movement for 2 days before his birth. He presented with apnea immediately after birth and his Apgar scores were very low, 1 and 2 points at 1 and 5 min, respectively. He was the second child born to non-consanguineous parents who were both 40 years old. The parents' first baby was also born at full term of spontaneous gestation 2 years before this case, and had weight appropriate for gestational age. This patient, however, weighed about 2420 g at birth. The amniotic fluid was clear, and the placenta, weighing 420 g, had no remarkable changes. A chest X-ray of the case showed marked pulmonary congestion and diffuse bilateral reticulogranular infiltrates in the lungs without evidence of cardiomegaly (Fig. 1) and an echocardiogram revealed that PV did not connect to the left atrium or CPV but an innominate vein-like small vein drained into the SVC, suggestive of TAPVD, supracardiac type. The patient could not have surgery because of his poor general condition. Although artificial ventilation after intubation and surfactant substitution therapy were performed, he died of hypoxemic respiratory failure, which was resistant to the intravenous administration of dopamine and bicarbonate and the inhalation of nitric oxide, 6 days after birth.

The blood cell counts and values of blood biochemistry showed thrombocytopenia (6.8 × 10^4^ /µL), high lactic acidemia (108 mg/dL) and severe hypoglycemia (4 mg/dL). Additionally, a venous blood sample when breathing 100% oxygen concentration in the incubator demonstrated elevated partial pressure of carbon dioxide (PaCO_2_; 73.6 mmHg), abd decreased base excess (−10.2 mEq/L) and pH (7.097), indicating marked respiratory acidosis. An autopsy was performed approximately 20 h after death, but the brain could not be examined due to the family's objections.

## PATHOLOGICAL FINDINGS

**Figure 2:**
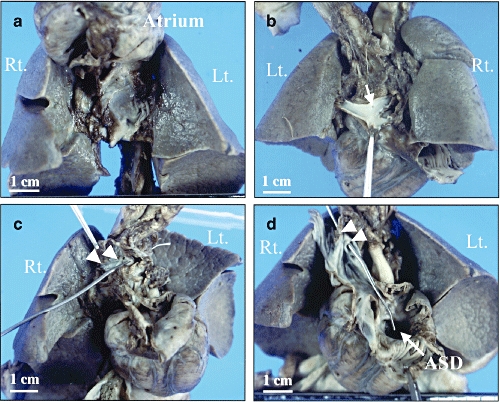
Gross findings of the heart and lungs. (**a**) On the anterior view of the lung with the heart displaced upward, no pulmonary veins (PV) returning to the left or right atrium are seen. (**b**) The posterior view of the lung reveals right and left inferior PV (IPV) from the lower lobes joining to form a blind confluence behind the left atrium with a tiny branch-like structure (arrow). No vertical veins draining into the innominate or portal vein are recognized. (**c,d**) The anomalous, small and hypoplastic right superior PV (SPV) (arrowheads, **c,d**) from the right upper lobe is identified (**c**), and apparently drains into the superior vena cava (SVC) (**d**), where the bloodstream is indicated by a metal sound (**c,d**). Partial and supracardiac type (type I) TAPVD is suggested. Although atrial septal defect (ASD, arrow) also coexists in the heart, the other major structural cardiac anomalies are absent. Moreover, there is no left SPV. Bar = 1 cm.

**Figure 3:**
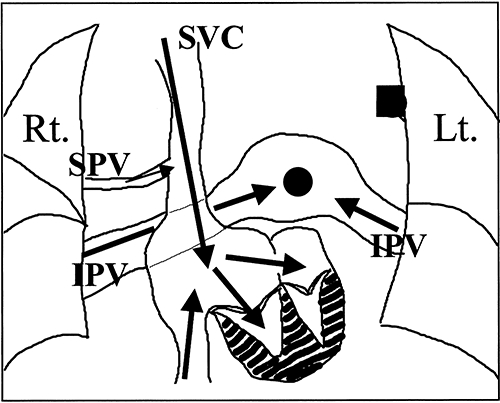
Schema of the anomalous heart. The present case has been diagnosed as partial and supracardiac type I TAPVD associated with ACPV (•) and atresia of left superior pulmonary vein (SPV) (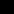). Atrial septal defect is also present. The bloodstream is indicated by arrows. IPV, inferior pulmonary veins; Lt., left; Rt., right; SVC, superior vena cava.

At autopsy, the baby measured approximately 45 cm in height and weighed 2420 g. An external examination showed anomaly of the right accessory auricle and a few needle marks on the extremities. In an internal examination of the thoracic cavity, markedly involuted thymus, weighing 1.5 g, was noted in the anterior mediastinum. The heart, weighing 21.5 g, showed a dilated and hypertrophic right ventricle and modest hypoplasia of the left ventricle without apparent PV returning to the left atrium (Fig. 2a), consistent with the findings of TAPVD. Although there were no PV draining into the right atrium or funiculi, reminiscent of an atretic fibrous band of CPV, between the atrium and pericardium (Fig. 2a) two IPV from the lower lobes of the lungs were seen to form a blind confluence behind the left atrium with a tiny branch-like structure (Fig. 2b), they did not connect with the epicardium or pericardial cavity. No vertical veins draining into the innominate or portal vein were noted. The diagnosis of ACPV was established based on these features. There was no apparent left superior PV (SPV) or atretic PV-like funiculus on a thorough gross examination, this case was considered to be associated with ACPV and likely atresia of one PV. As to the right SPV, an anomalous, small and hypoplastic PV (Fig. 2c,d) from the right upper lobe was identified (Fig. 2c) that drained into the SVC (Fig. 2d), indicating that this case was partial and supracardiac type I TAPVD, according to Darling's classification.^5^ Atrial septal defect (ASD) and persistent ductus arteriosus (PDA) were present. However, other major structural cardiac anomalies, such as single ventricle, single atrium and anomalies of truncus arteriosus, were absent. The schema of this anomalous heart is summarized in Figure 3. The left and right lungs weighed 25.4 g each and were firm in consistency. The cut surfaces of the congested lungs after formalin fixation showed modestly thickened interlobular septum and hilum without evidence of cystic changes. An internal examination of the abdominal cavity exhibited clear yellow peritoneal fluid (100 mL) and markedly atrophic and hemorrhagic adrenal glands, weighing 4.4 g combined, without other extracardiac malformations including visceral situs inversus or polysplenia.

**Figure 4:**
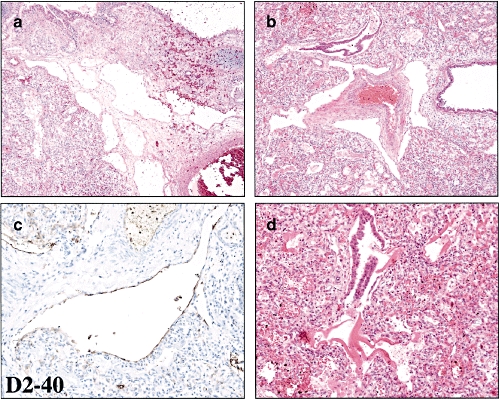
Histological findings of the secondary pulmonary lymphangiectasis. (**a–c**) Low-power views of the lung demonstrate diffuse and conspicuous lymphatic dilation of the interlobular (**a**) and peribronchial (**b**) areas. They are invariably lined by flattened lymphatic endothelium (HE stain), which is immunohistochemically positive for D2-40 (**c**). (**d**) Many mature-looking alveolar spaces are collapsed, and the alveolar walls are close to each other with deposits of hyaline membrane along the bronchioli to the alveolar ducts (HE stain).

**Figure 5:**
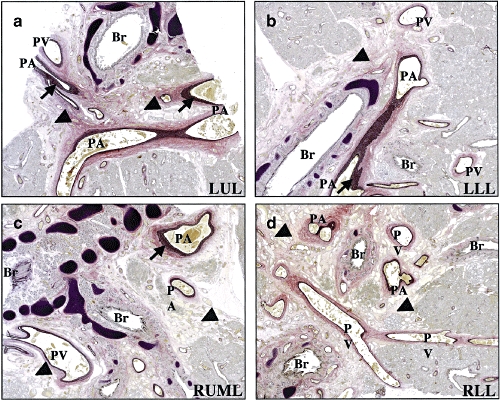
Histological findings of each of the lung lobes in the hilar areas. (**a–d**) On scan magnifications, the pulmonary arteries (PA) do not manifest intimal hyperplasia, but slight to mild and focal medial hypertrophy (arrows) in each of the bilateral lobes (Elastica van Gieson stains), compared with those of another neonate autopsy case without any evidence of cardiac anomalies. The left superior pulmonary vein (SPV) vessels on the peripheral side can be identified in the left upper lobe (LUL) (**a**), and we thus finally made a conclusive diagnosis of atresia of the left superior vena cava (SPV). However, the atretic left SPV on the central side cannot be recognized. There are no clear distinctions of the pulmonary veins (PV) in size or shape between this present case and the control case. Moreover, a mild to moderate and diffuse secondary PL is also noted (arrowheads). Br, bronchus; LLL, left lower lobe; RLL, right lower lobe; RUML, right upper and middle lobes.

A histological examination of the heart revealed hypertrophy of the right cardiac muscle, but no subendocardial inflammation or infarction was evident. The lungs showed diffuse and moderate lymphatic dilation in the interlobular (Fig. 4a), hilar (Fig. 5a–d), peribronchial (Fig. 4b) and focally, subpleural areas. The dilated lymphatic channels were invariably lined with flattened endothelium, which was immunohistochemically positive for D2-40 (Nichirei Bioscience Co., Tokyo, Japan; 1:1 dilution) (Fig. 4c), CD31 (DakoCytomation Co., Tokyo, Japan; 1:20 dilution) and CD34 (IMMUNOTECH, Marseille, France; 1:50 dilution). No CD68-positive (Dako; 1:100) foreign body type histiocytes were seen in these cystically dilated lesions. No lymphangiectasis was found around the mediastinum or the intra-abdominal organs. The diagnosis of secondary pulmonary lymphangiectasis (PL) due to congenital cardiovascular anomalies, including TAPVD and ACPV, Noonan classification group 2^17^ was established based on these features. Most of the mature-looking alveolar spaces were collapsed, and the alveolar walls were close to each other with frequent deposits of hyaline membrane along the bronchioli or alveolar ducts (Fig. 4d). Moreover, the pulmonary arteries (PA) did not manifest intimal hyperplasia, but did show slight to mild and focal medial hypertrophy in each of the bilateral lobes (Fig. 5a–d), displaying a clear contrast with Elastica van Gieson (EVG) staining compared to those of the other full term neonate autopsy cases without any evidence of cardiac anomalies. Despite no evidence of a left SPV-like funiculus on gross findings, left SPV vessels on the peripheral side could be identified microscopically in the parenchyma of the left upper lobe (Fig. 5a), hence, we finally made a conclusive diagnosis of atresia, not aplasia, of the left SPV. Histological examination of each lobe showed there were no clear distinctions in the size or shape of PV in this present case and the control case. Additionally, atretic left SPV and CPV in the centre could not be identified.

The markedly involuted thymus showed advanced lymphocyte depletion without a starry-sky pattern accompanied by aggregated Hassall's corpuscles with a mild inflammatory infiltrate of neutrophils. Pseudofollicular cysts were present in the definitive cortex of severely atrophic adrenal glands. These findings were considered likely due to severe stress during intrauterine life and after birth.

## DISCUSSION

ACPV is an extremely rare congenital heart disease with a very poor prognosis, first described by Lucas *et al*. in 1962,^10^ demonstrating clinical manifestations of aggressive cardiorespiratory failure immediately after birth, cyanosis, acidosis and death.^1^,^2^,^6^–^9^ Although early clinical recognition and immediate surgical treatment are essential, it is very difficult to establish an antemortem diagnosis of ACPV, and thus it results in an adverse clinical outcome.^2^,^7^–^9^ The definitive diagnosis can be made by cardiac catheterization or during surgery, however, determining it at autopsy accounts more than 40% of reported cases.^2^,^18^ Nevertheless, due to the recent advances in neonatal care, the outcome of the patients has improved. Dudell *et al*. reported three cases of corrective surgery; despite the required continued postoperative support with extracorporeal membrane oxygenation due to pulmonary hypertension and severe pulmonary parenchymal disease, two patient survived and were discharged from the hospital.^2^

ACPV can be considered an advanced form of TAPVD, which is a much more common entity and whose etiology is probably early atresia of the CPV.^4^,^6^ In contrast, late atresia after involution of the embryonic collaterals to the systemic veins such as azygos and/or hemiazygos veins leads to ACPV.^1^,^2^,^6^–^11^,^13^ The PV of ACPV do not communicate with either the left atrium or the systemic venous system, but join between them with a very closed and blind confluence.^1^,^2^,^6^–^11^,^13^ Funiculi, reminiscent of an atretic fibrous band of CPV, are rarely identified between the atrium and pericardial cavity in cases of ACPV.^1^,^19^ Although in the present case there are no apparent atretic fibrous bands, it is presumed that the tiny branch-like structure in the closing IPV (Fig. 2b) might have connected to the CPV before the ACPV occurred. Only a few cases of TAPVD associated with ACPV are reported in the literature.^2^,^19^,^20^ The cases, however, varied in TAPVD types and associated anomalies, for example, infracardiac type (type III) TAPVD accompanied by not only ACPV with an atretic fibrous band, but also atresia of paraesophageal abnormal PV connection in a Japanese case,^19^ supracardiac type (type I) TAPVD associated with ACPV in another Japanese case,^20^ and supracardiac type (type I) TAPVD associated with not only ACPV but also aplasia of the right and left superior PV in an American case.^2^ Thus, ACPV cases, including ours, can have wide varieties of features sometimes closely related with TAPVD, and the presence of TAPVD and ACPV in the same siblings may indicate the genetic relationship between two.^2^ Furthermore, although TAPVD cases very rarely accompany ACPV, ACPV with or without TAPVD may be more common than generally considered, because those clinical or radiological features are very difficult to distinguish.^1^,^2^,^8^ ACPV is the only occasion where congestive cardiac failure occurs without cardiomegaly, this contrasts with TAPVD where a large heart is commonly present.^1^,^4^ It is well known that the increased pulmonary pressure in ACPV causes a pressure overload on the right ventricle and hypertrophy occurs,^1^,^2^ but, interestingly, no cardiomegaly is detected clinicopathologically. This is because atrophy or hypoplasia of the left ventricle could coexist with hypertrophy of the right one, as in the current case. However, in addition, differential diagnoses of other cardiac anomalies should be considered, including pulmonary vein stenosis, cor triatriatum, congenital mitral stenosis, or persistent fetal circulation. Echocardiography, particularly two-dimensional sector scanning, might prove helpful in distinguishing these conditions.^8^ If echocardiographic examinations cannot be performed, a chest radiography showing pulmonary congestion without cardiomegaly in combination with an electrocardiogram revealing right ventricular hypertrophy and right axis deviation strongly suggest ACPV.^1^,^8^ The X-ray in the present case (Fig. 1) showed marked pulmonary congestion and diffuse bilateral reticulogranular infiltrates as a partial result of dilated pulmonary lymphatic channels with an almost normal-sized heart, resembling the findings previously reported,^1^,^19^,^20^ however, the electrocardiogram of the case has not been available. Nevertheless, a tentative diagnosis of ACPV should be borne in mind whenever a clinician encounters a neonate with severe cyanosis and acidosis which are unresponsive to oxygen therapy and resistant to early medical management.

Although the prognosis of ACPV is usually fatal and survival beyond 4 weeks is indeed very rare, only a few neonates without effective surgical management may survive because of the presence of PV outlet in the various forms, such as bronchial venous circulation, minor PV communication, pulmonary arteriovenous malformation and dilated lymphatic channels.^8^,^12^,^14^,^15^ One unusual ACPV case has been reported in a 6-month-old infant; this child had suffered from complex congenital heart disease and visceral heterotaxy associated with anomalous drainage of the right SPV.^8^ As to the current case, survival of 6 days is not exceptional, however, the fact that anomalous right SPV drained into the SVC, might be uncommon. On the other hand, this anomalous right SPV was smaller in size, and probably could function less than the IPV, given that histopathological examination of the lungs showed no remarkable difference between right and left (Fig. 5a–d) (i.e. no congestion, medial hypertrophy of pulmonary arteries, or pulmonary lymphangiectasis (PL), demonstrating signs of pulmonary arterial hypertension).^16^,^17^ Nevertheless, as described above, there could be unidentifiable minor PV outlets including the outflow from the closing IPV or left SPV, the circulation system of the bronchial arteries and veins, and dilated pulmonary lymphatic flows, as an assistant circulation system of this anomaly. There is the possibility that this assistant system might not lead to significantly distinct findings between the right and left lung lobes. Many ACPV cases, including ours, have reported the complications of secondary PL, whereas the change to pulmonary vascular vessels, such as marked medial hypertrophy or intimal hyperplasia, is inconspicuous.^7^–^9^ It is very likely that PL could be more easily reflected in pulmonary arterial hypertension in the agonal phase. PL is characterized by a severely disturbed pulmonary gas exchange leading to a poor prognosis because of the compression of the alveolar tissues and reduction of the alveolar spaces.^16^ The present case displays not only PL but also hyaline membrane disease in the congested lungs (Fig. 4), and the cause of death in this patient is considered to be cardiorespiratory failure.

In summary, we report a unique neonate autopsy case of TAPVD type I, in which anomalous and relatively small right PV only drained into the SVC, associated with ACPV of left and right IPV in a blind confluence, and atresia of left SPV, featuring one series and a wide variety of PV atresia. Secondary PL was identified in the histopathological examination. Although TAPVD with ACPV is an extremely rare anomaly, we should be aware that ACPV is one of the possible causal factors in the development of severe pulmonary symptoms in neonates.
